# Pachymaran alleviates fat accumulation, hepatocyte degeneration, and injury in mice with nonalcoholic fatty liver disease

**DOI:** 10.1515/med-2025-1241

**Published:** 2025-07-24

**Authors:** Hong Yu, Min Wan, Hong Li, Xing Liu

**Affiliations:** Medical Laboratory, Wuhan Hospital of Traditional Chinese Medicine, Wuhan, Hubei, 430050, China; Medical Laboratory, Wuhan Hospital of Traditional Chinese Medicine, No. 303, Sixin Avenue, Hanyang District, Wuhan, Hubei, 430050, China

**Keywords:** pachymaran, nonalcoholic fatty liver disease, adenylate-activated protein kinase, liver, fat

## Abstract

**Background:**

Nonalcoholic fatty liver disease (NAFLD) is characterized by excessive hepatic fat accumulation and is closely associated with obesity, diabetes, and hyperlipidemia.

**Objectives:**

This study explores the effects of pachymaran on NAFLD induced by a high-fat diet (HFD) in a murine model.

**Methods:**

Thirty male C57BL/6 mice were allocated into five groups: normal diet (ND), NAFLD, and high-, medium-, and low-dose pachymaran (200, 100, and 50 mg/kg, respectively). All groups except the ND were fed a HFD to induce NAFLD. The pachymaran groups received daily intragastric pachymaran for eight weeks. Post-treatment, liver weight were recorded, serum indices assessed, and hepatic pathology evaluated via histological and Oil Red O staining. Adenylate-activated protein kinase (AMPK) gene expression was analyzed through western blotting.

**Results:**

The body weight and liver gain (87.8 and 23.0%) in the high-dose pachymaran group were significantly less than those (154.2 and 82.0%) in the NAFLD group (*P* < 0.05). Fat content and serum indices improvements correlated with increased pachymaran doses. Histological analyses indicated significant alleviation of hepatocyte hypertrophy and ballooning steatosis in treated groups. Oil Red O staining confirmed a substantial decrease in hepatic lipid droplets, and western blot results indicated a significant increase in AMPK phosphorylation following treatment (*P* < 0.05).

**Conclusions:**

Pachymaran effectively mitigated fat accumulation, hepatocyte degeneration, and injury in mice with diet-induced NAFLD, likely through modulation of the AMPK pathway.

## 1 Introduction

Nonalcoholic fatty liver disease (NAFLD) is a pathological condition defined by excessive lipid accumulation in liver cells not caused by alcohol, drugs, viral infections, autoimmunity, or other specific hepatotoxic factors [1]. Initially marked by simple steatosis, NAFLD can progress to nonalcoholic steatohepatitis (NASH), characterized by inflammation and, in 10–20% of cases, liver fibrosis, forming high-risk steatohepatitis [2,3]. NASH may further evolve into cirrhosis and eventually liver cancer. Globally, NAFLD affects approximately 25% of the population, with the highest prevalence rates in South America and the Middle East, each exceeding 30% [4]. Remarkably, the prevalence in China is increasing at twice the rate of developed countries, predicted to be the fastest growing by 2030 [5]. This underscores the urgent need for effective NAFLD interventions.

Current therapeutic strategies for NAFLD focus on reducing body fat and targeting lipid metabolism [6,7]. However, the few available drugs have uncertain safety and efficacy profiles. Standard treatments involve weight loss alongside lipid-lowering and anti-inflammatory liver-protecting medications that address metabolic disturbances [8]. Prolonged use of these pharmaceuticals can lead to significant side effects, highlighting the necessity for safer, functional molecules that can ameliorate NAFLD.

Poria cocos, an edible medicinal fungus used in traditional Chinese medicine for thousands of years, contains pachymaran, known for its antitumor, antiaging, antidiabetic, anti-inflammatory, and antihemorrhagic fever effects [9,10]. Pachymaran is synthesized from natural sources through processes including ethanol alkalization, initial chloroacetic acid etherification, and secondary base etherification [11]. Pachymaran can reduce the increase of serum alanine aminotransferase and aspartate transaminase contents in mice caused by carbon tetrachloride and improve the regeneration ability of liver resection in rats. In addition, Pachymaran can increase the activities of antioxidant enzymes such as superoxide dismutase, catalase, and glutathione peroxidase and reduce the contents of peroxides ROS and MDA [1]. Previous studies suggest that pachymaran offers protective benefits against liver oxidative damage and therapeutic potential for liver inflammation and cell apoptosis [12], yet its role in NAFLD requires further exploration.

Research indicates the involvement of the adenylate-activated protein kinase (AMPK) pathway in NAFLD [13]. Activation of AMPK enhances the activity of downstream transcription factors like peroxisome proliferator-activated receptor alpha (PPAR-α) and the α subunit of peroxisome proliferator-activated receptor-γ coactivator-1 (PGC-1α), increasing oxidative phosphorylation of fatty acids and promoting liver energy metabolism [14]. In this study, we used a C57BL/6 mouse model divided into normal diet (ND), NAFLD, and various pachymaran-treated groups to investigate the effects of pachymaran on NAFLD and its potential molecular mechanisms. We assessed liver metabolism and pathological alterations in NAFLD mice treated with pachymaran, exploring its effects through histological staining, Oil Red O staining, and western blot analysis of AMPK gene expression.

## 2 Materials and methods

### 2.1 Establishment of animal model

A murine model of NAFLD was established using 7-week-old male Specific Pathogen Free KM mice (16–18 g). All mice were housed in standard cages with a light/dark cycle of 12 h at a temperature of 18–25°C and a relative humidity of 65–70%. Mice were randomly allocated to control (ND, *n* = 6) and high-fat diet (HFD, *n* = 24) groups according to a random number table, with no initial significant difference in body weight. HFD feed: 60% fat, 20% carbohydrates, 20% protein. Mice in the HFD group were fed for 13 weeks to induce the establishment of NAFLD. The mice in the ND group were fed with ND for 13 weeks. After the model was established, whether the NAFLD model was successfully established was evaluated through the observation of pathological changes, body and liver weights, body fat percentage, and serological indices. All procedures were performed in compliance with the Guide for the Care and Use of Laboratory Animals by the NIH, approved by the ethical committee of Wuhan Hospital of Traditional Chinese Medicine (Approval No. 2023-009).

### 2.2 Pachymaran treatment

Pachymaran extract (containing pachymaran ≥90%) was obtained from Sichuan Vicchi Biotechnology Co. Ltd. (Chengdu, China). The HFD group was subdivided into four groups: an untreated NAFLD group and low- (50 mg/kg), medium- (100 mg/kg), and high-dose (200 mg/kg) pachymaran groups. Pachymaran was administered intragastrically daily for 8 weeks. Control mice received an equivalent volume of saline. At the endpoint, mice were euthanized in a CO_2_ chamber, followed by cervical dislocation. Tissue samples were collected for subsequent analyses.

### 2.3 Body fat and organ ratio assessment

Post-treatment, mice were weighed and put into the body fat meter (E1452120, Rheinstetten, Germany) according to different groups. The fat content, lean meat content, and fluid content in mice were detected. Then, the mice were euthanized. The white adipose tissue from various sites and the liver were weighed, and the obesity index was calculated as the ratio of total white adipose tissue to body mass × 100.

#### 2.3.1 Hematoxylin and eosin staining

Part of the liver tissue was fixed with 4% paraformaldehyde, dehydrated with alcohol, made transparent with xylene, embedded in paraffin, and stone wax sections were prepared. The slices were stained with hematoxylin for approximately 30 min, stained with eosin staining solution for 1 min, rinsed, dehydrated, sealed with transparent and neutral gum, and morphologically observed.

#### 2.3.2 Masson staining

Remove the frozen paraffin pieces and leave them at room temperature for 20 min to rewarm. The sections were stained with Masson’s trichrome and observed under a microscope (IX-51, Olympus).

#### 2.3.3 Oil Red O staining

The frozen slices of liver tissue were prepared, and the slices were incubated with 100% isopropyl alcohol for 5 min, then with 0.5% Oil Red O solution for 20 min, cleaned with 85% isopropyl alcohol solution for 3 min, washed, stained with hematoxylin for 1 min, washed, sealed with tablet, and observed for fat staining.

### 2.4 Serological analysis

Blood samples were collected for serum separation and stored at –80°C. Serum triglycerides, total cholesterol, low-density lipoprotein (LDL), and high-density lipoprotein (HDL) levels were measured using kits from Nanjing Jiancheng Bioengineering Institute, China.

### 2.5 Western blot analysis

Proteins from liver tissues were extracted and separated using 10% sodium dodecyl sulfate-polyacrylamide gel electrophoresis. A Micro BCA Protein Assay Kit (Pierce, Rockford, IL, USA) was used to quantify protein concentrations in the supernatants. Equal amounts (20 µL) of protein were loaded onto the gels, and the separated proteins were transferred to polyvinylidene fluoride membranes. The membranes were blocked in tris buffered saline containing 5% Tween 20 and 5% skim milk at room temperature for 1 h and incubated overnight at 4°C with a primary antibody (p-AMPK [Cat#2535S, 1:1,000; CST, Danvers, MA, USA], AMPK [Cat#2532S, 1:1,000; CST], and CaMKKβ [Cat#DF4793, 1:1,000; Affinity Biosciences, Cincinnati, OH, USA]). β-actin (ab8245) (mouse, 1:1,000; Abcam, Cambridge, England) was used as an internal control. Primary antibodies were detected using a secondary anti-mouse or anti-rabbit IgG antibody coupled with HRP (1:5,000, ab6728, Abcam, Cambridge, England). Target proteins were visualized using an EZ-ECL chemiluminescence detection kit (Pierce, Rockford, IL, USA).

### 2.6 Immunohistochemistry

The liver tissue of the mice in each group was sliced, and protein expression was observed using immunohistochemistry. Liver tissues were fixed with 4% paraformaldehyde and embedded in paraffin. The samples were then sectioned into slices with a thickness of 6 μm, which were then attached to slides, washed, and incubated with 3% H_2_O_2_ in methanol for 10 min to block endogenous peroxidase activity. The sections were washed with phosphate buffered saline (PBS) and incubated overnight at 4°C with a primary antibody (p-AMPK [Cat#2535S, 1:100; CST, Danvers, MA, USA], AMPK [Cat#2532S, 1:100; CST], and CaMKKβ [Cat#DF4793, 1:100; Affinity Biosciences, Cincinnati, OH, USA]). Subsequently, the sections were washed three times in PBS for 5 min and incubated with biotinylated anti-mouse or anti-rabbit (1:1,000; ab6728, Abcam, Cambridge, England) at room temperature for 20 min. The sections were washed with PBS, and avidin–horseradish peroxidase complex was added (SABC Kit; Bost, Wuhan, China). 3,3-Diaminobenzidine was used as a chromogen. The positive cells were visualized under a light microscope.

### 2.7 Statistical analysis

Data were analyzed using GraphPad Prism 8.0 software (GraphPad Software, Boston, MA, USA). Results are presented as the mean ± standard deviation. Statistical differences between the groups were evaluated using one-way analysis of variance, followed by the *post hoc* Tukey multiple comparison test. Trends with dose variation were assessed using repeated measures analysis of variance. Statistical significance was set at a *P* < 0.05.

## 3 Results

### 3.1 Effects of pachymaran on body and liver weights in mice

**Figure 1:**
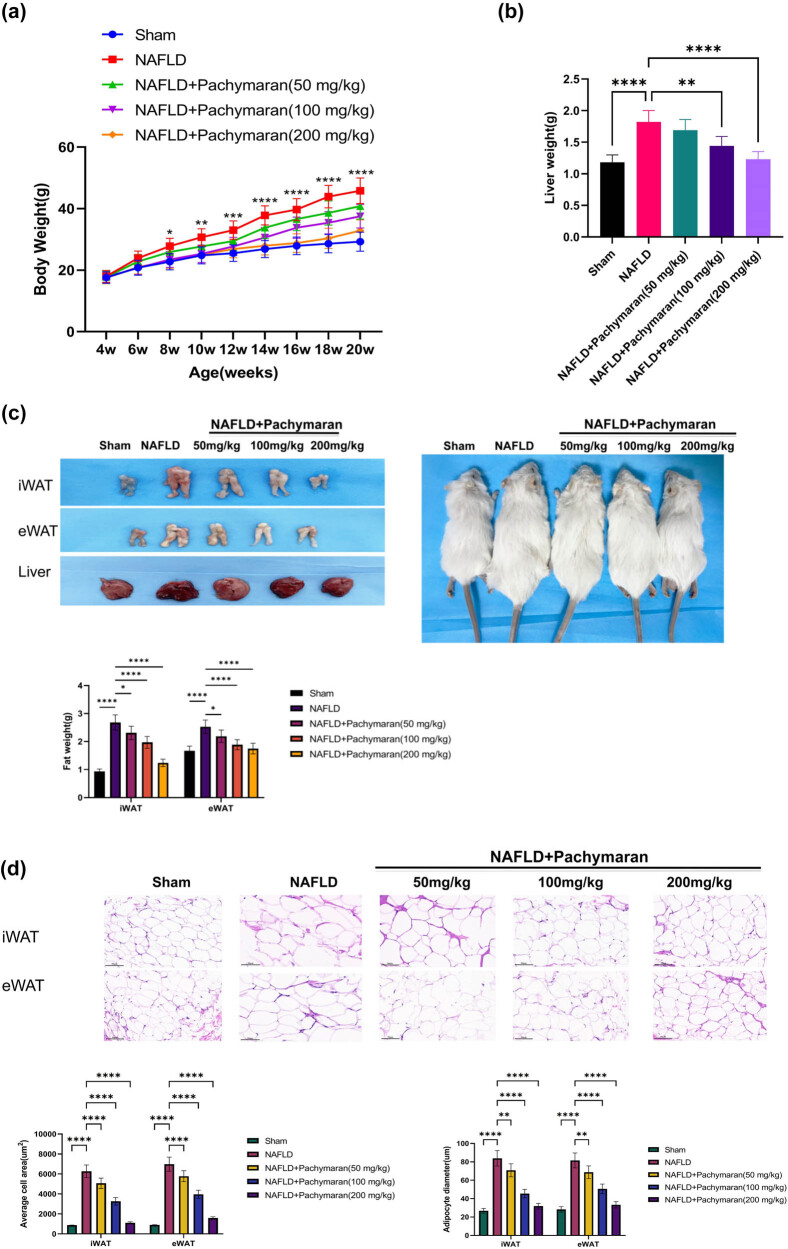
Pachymaran inhibited weight gain and liver fat accumulation in NAFLD mice. (a) Growth curves of mice during treatment. (b) Liver weight in the eighth week. (c) Morphological pictures of inguinal white adipose tissue (iWAT), epididymal white adipose tissue (eWAT), liver, and mice, and the relative fat mass at week 8. (d) iWAT and eWAT sections stained with hematoxylin and eosin visualized under a 400× light microscope, and the average area and diameter of adipocytes in iWAT and eWAT. *n* = 3, *P < 0.05, **P < 0.01, ***P < 0.001, ****P < 0.0001. (magnification, 200×).

Beginning from the second week, the average body weight of the NAFLD group was significantly higher than that of the ND group (*P* < 0.05). In contrast, pachymaran-treated mice displayed significantly reduced body weight compared to the NAFLD group, with more notable reductions observed at higher dosages (Figure 1a). Similarly, while liver weight in the NAFLD group showed a significant increase by the eighth week, those in the pachymaran-treated groups decreased in a dose-dependent manner (Figure 1b). Compared with the sham group, mice in the NAFLD group had increased subcutaneous and abdominal fat. Pachymaran treatment effectively reduced fat accumulation, progressively returning to the size in healthy controls as the dosage increased (Figure 1c). Furthermore, both the area and diameter of adipocytes in inguinal white adipose tissue and epididymal white adipose tissue were significantly larger in the NAFLD group compared to controls. However, these measures significantly decreased in the pachymaran-treated groups, correlating inversely with the increasing drug concentrations (Figure 1d).

### 3.2 Effect of pachymaran on body fat and serological indicators in mice

**Figure 2:**
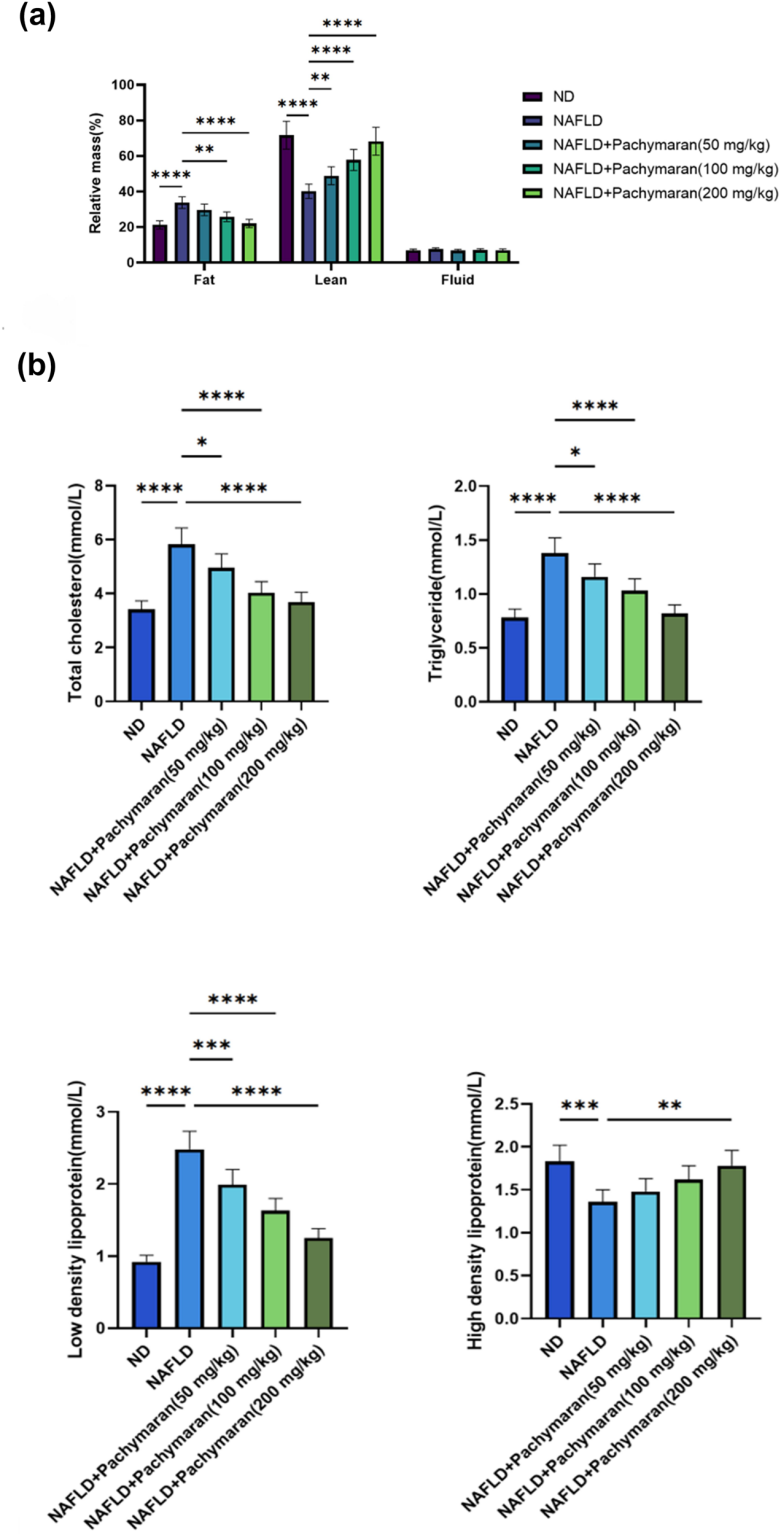
Pachymaran decreased body fat percentage and serological indices of mice NAFLD. (a) The relative ratio of systemic fat, lean, and liquid mass at week 8. (b) Changes in serum TG, TC, LDL, and HDL at week 8. *n* = 3, *P < 0.05, **P < 0.01, ***P < 0.001, ****P < 0.0001.

Body composition analysis *in vivo* revealed that mice with NAFLD exhibited increased fat tissue content. These adverse effects were progressively mitigated as the dosage of pachymaran increased (Figure 2a). Compared to the ND group, serum levels of total cholesterol (TC), triglycerides (TG), and low-density lipoprotein cholesterol (LDL-C) were significantly elevated in the NAFLD group. However, with increasing doses of pachymaran, these levels gradually decreased. Conversely, high-density lipoprotein cholesterol (HDL-C) levels, which were lower in the NAFLD group, increased with higher pachymaran dosages (Figure 2b).

### 3.3 Effect of pachymaran on hepatocyte steatosis

**Figure 3:**
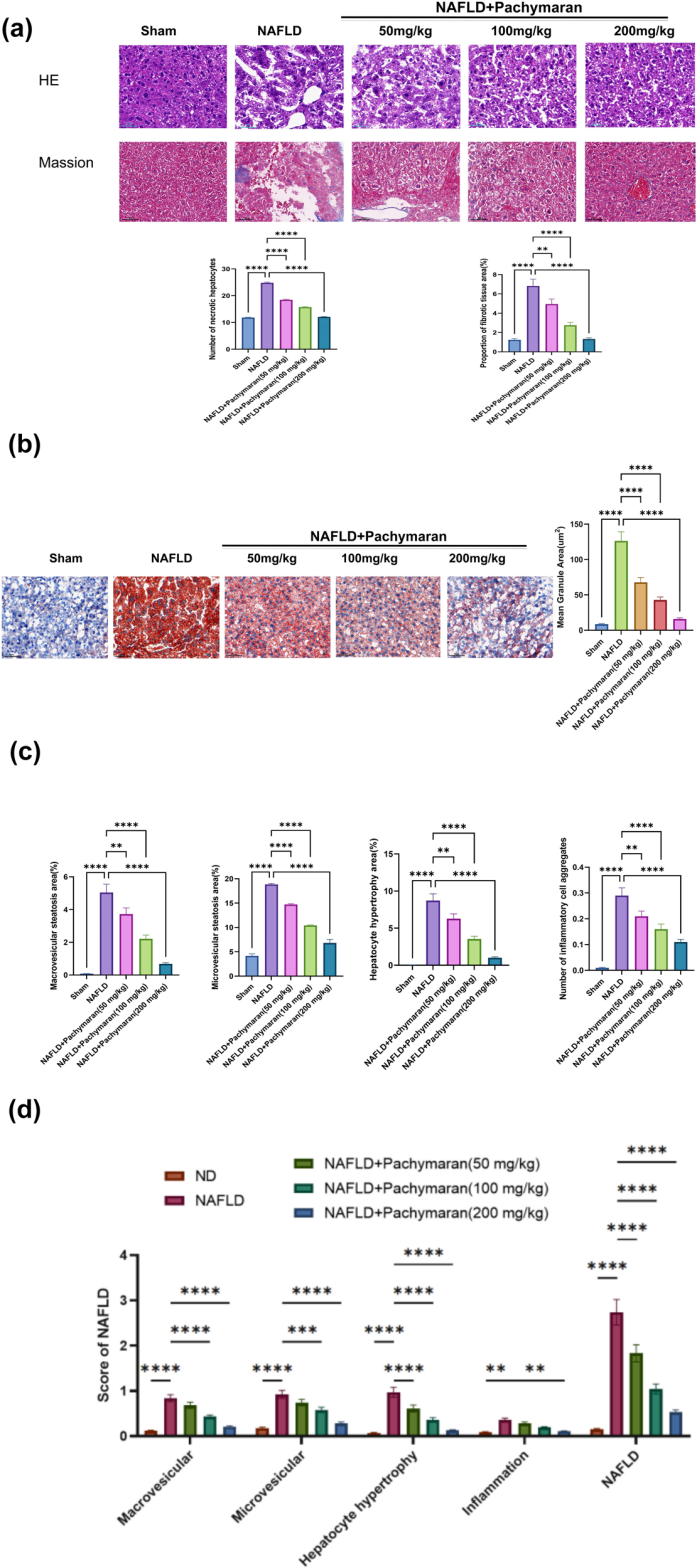
Pachymaran prevented liver injury in mice with NAFLD. (a) Morphological images of liver tissue stained with hematoxylin and eosin and Masson’s trichrome. (b) Morphological images of liver tissue stained with Oil Red O (100×). (c) Percentage of hepatic bullous steatosis, vesicular steatosis, hypertrophic area of hepatocytes, and number of inflammatory cell aggregates in the liver tissue. (d) Hepatic bullous steatosis, vesicular steatosis, hypertrophic area of hepatocytes, and inflammatory cell aggregate scores and total NAFLD score. *n* = 3, *P < 0.05, **P < 0.01, ***P < 0.001, ****P < 0.0001. (magnification, 200×).

Histological analyses were conducted on liver tissues stained with hematoxylin and eosin and Masson’s trichrome to assess cellular structure and fibrosis. In the NAFLD group, hepatocytes exhibited edema and balloon degeneration, with instances of single or scattered necrosis and perihepatocytic infiltration by lymphocytes and macrophages. Minor fibrotic changes were noted within the hepatic lobules, though no bridging fibrosis was observed in lobular or perisinusoidal spaces. Treatment with pachymaran ameliorated these pathological changes, with more pronounced improvements at higher doses (Figure 3a).

Oil Red O staining was utilized to visualize fat accumulation within liver tissues. The NAFLD group displayed large lipid droplets and significant steatosis, whereas pachymaran treatment effectively reduced fat accumulation, with greater efficacy at higher concentrations (Figure 3b).

The severity of NAFLD was further quantified using an established scoring system [15], evaluating macrovesicular steatosis, microvesicular steatosis, hepatocyte hypertrophy, and inflammatory cell aggregation. The NAFLD group exhibited significant steatotic changes and increased aggregation of hypertrophic hepatocytes and inflammatory cells. Conversely, pachymaran treatment led to a dose-dependent reduction in these parameters (Figure 3c). Accordingly, NAFLD scores increased in the untreated group but decreased in the pachymaran-treated groups (Figure 3d).

### 3.4 Effect of pachymaran on the AMPK signaling pathway in mouse liver

**Figure 4:**
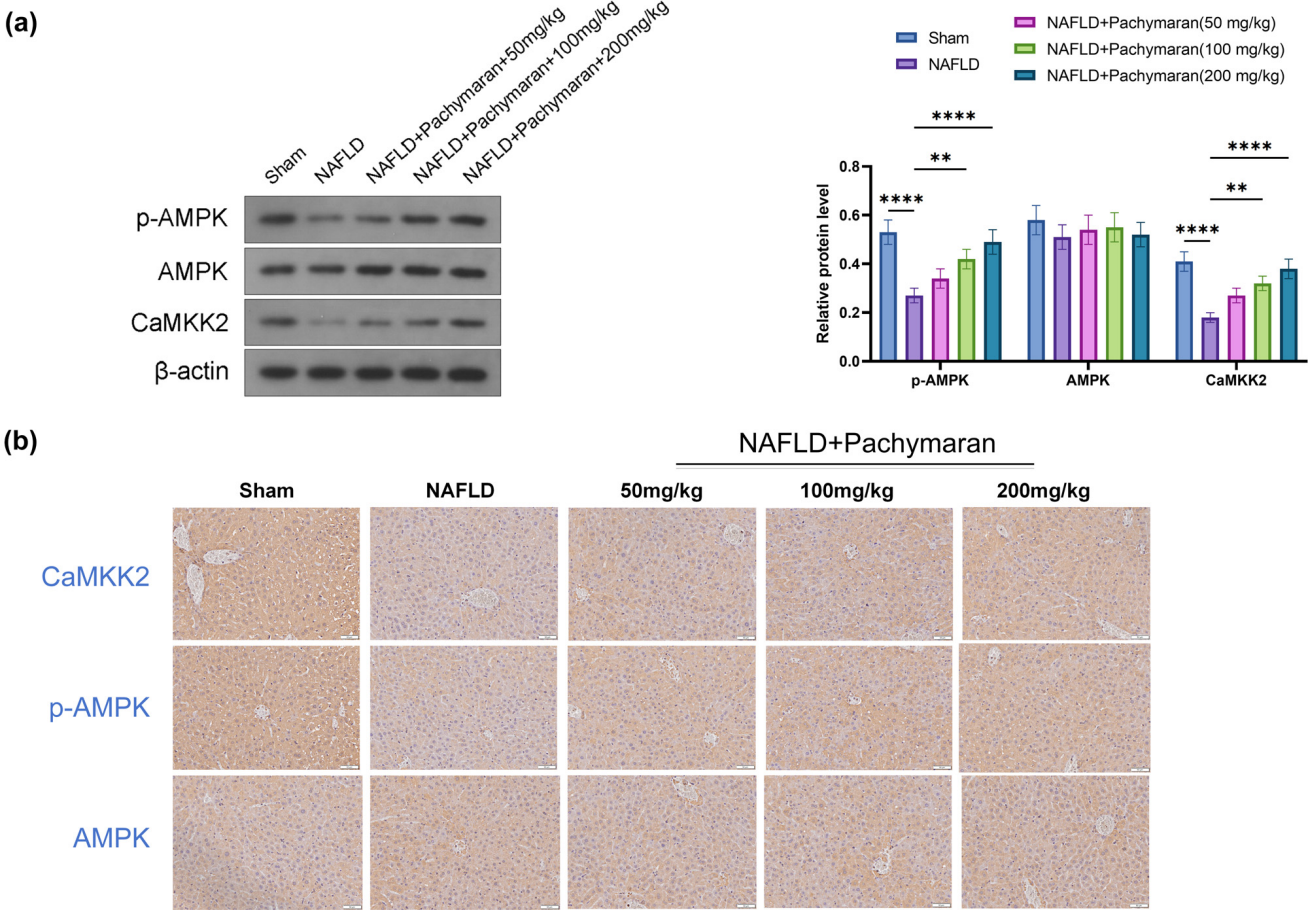
The p-AMPK, AMPK, and CaMKK2 levels in mice with NAFLD. (a) Western blot reflected the expression of p-AMPK, AMPK, and CaMKK2. (b) Immunohistochemistry staining reflected the expression of p-AMPK, AMPK, and CaMKK2. *n* = 6, *P < 0.05, **P < 0.01, ***P < 0.001, ****P < 0.0001.

Western blot and immunohistochemistry analysis revealed a significant reduction in the levels of CaMKK2 and phosphorylated AMPK (p-AMPK) in the livers of mice from the NAFLD group. In contrast, mice treated with pachymaran displayed increased expression of both CaMKK2 and p-AMPK. Notably, this enhancement in expression was dose-dependent, with the highest concentrations of pachymaran producing the most pronounced effects (Figure 4a and b).

## 4 Discussion

NAFLD is a chronic metabolic disorder characterized by lipid deposition and a nonspecific inflammatory response in the liver, with complex etiology and pathogenesis. Traditional treatments for NAFLD primarily involve lifestyle and dietary changes, which can be challenging to maintain in a fast-paced society. Consequently, pharmacological interventions are increasingly viewed as essential. Pachymaran, known for its atherosclerosis mitigation properties in HFD induced models by reducing inflammatory factors and lipid levels [10], is yet to be thoroughly studied for its effects on NAFLD.

Obesity, a key risk factor for NAFLD, is effectively modeled by HFD in mice to mimic the pathophysiological impact of metabolic factors. In our study, a mouse model of NAFLD induced by HFD was established to explore the therapeutic effects and possible mechanism of action of pachymaran on NAFLD. In the present study, mice were fed HFD to induce NAFLD, and pachymaran prevented this weight gain. Lipid metabolism disorders are important in the pathogenesis of NAFLD [16]. TC is also closely associated with the development and occurrence of NAFLD. Free TC is a toxic lipid molecule that directly increases the production of reactive oxygen species by destroying mitochondria, promoting inflammation, promoting the inflammatory pyroptosis of hepatocytes, damaging the endoplasmic reticulum, and triggering endoplasmic reticulum stress, which ultimately leads to hepatocyte apoptosis [17]. The deposition of LDL particles in the liver can activate Kupffer cells and hepatic astrocytes in the liver, induce the release of inflammatory cytokines such as TNF-α and IL-6, and promote the expression of fibrosis factors, thus resulting in liver tissue injury, inflammation, and fibrosis [18]. Additionally, TG is usually processed in the liver; however, when the body ingests excess TG that exceeds the processing capacity of the liver, it is deposited in the liver [19]. Long-term and excessive TG deposition causes hepatocyte steatosis, leading to pathophysiological changes such as oxidative stress, endoplasmic reticulum stress, inflammation, and hepatocyte apoptosis, which gradually progress to hepatitis, liver fibrosis, and even liver cancer in the presence of other factors [20]. Therefore, lowering TG levels is an important strategy for NAFLD treatment. In the present study, pachymaran reduced the serum levels of TG, TC, and LDL-C (Figures 1 and 2). In addition, we found that pachymaran reduced body fat percentage. Our findings demonstrate that pachymaran significantly reduced serum levels of TG, TC, and LDL-C, suggesting its potential in reducing hepatocyte lipid deposition and subsequent cellular damage.

Histologically, NAFLD is predominantly characterized by hepatic steatosis, where nutrient intake exceeds the secretory capacity of the liver [21]. Pachymaran treatment not only reduced liver weight but also improved liver morphology, as evidenced by decreased hepatocyte size and bulla steatosis with dose-dependent neat cell arrangement and diminished lipid droplets observed via Oil Red O staining (Figures 1b and 3). These results underscore pachymaran’s efficacy in reducing lipid deposition and preventing hepatocyte steatosis.

At the molecular level, the *AMPK* signaling pathway plays a crucial role in energy metabolism and fatty acid oxidation [22]. Activation of the *AMPK* pathway inhibits lipogenesis in mice with NAFLD [23]. To further explore the mechanism of action of pachymaran in improving lipid metabolism disorders in the liver, we assessed the expression and phosphorylation of proteins involved in the *AMPK* pathway in the liver. Our study revealed that pachymaran enhanced *AMPK* phosphorylation in the liver after treatment, which may explain the inhibition of fatty acid synthesis and reduction in fat content, thus alleviating NAFLD. In addition, Ca^2+^-mediated activation of *CaMKKβ* is a common mechanism by which metabolism-related hormones induce *AMPK* activation [24]. A previous study showed that *CaMKKβ* was involved in the activation of *AMPK* when vascular endothelial growth factor B inhibited adipogenesis in mice with NAFLD [25]. In the present study, the expression of *CaMKKβ* in mouse liver tissues was also changed after treatment with pachymaran, which may be one of the mechanisms through which pachymaran exerts its therapeutic effects through *AMPK*. However, further experiments are needed to confirm these molecular interactions and their relevance to NAFLD treatment.

Limitations of this study include the need to determine the optimal therapeutic concentration of pachymaran and to validate these findings in cellular models, as well as to comprehensively establish whether pachymaran’s therapeutic effects are primarily mediated through the *AMPK* pathway.

In conclusion, pachymaran exhibits promising potential in inhibiting fat accumulation and alleviating cellular damage in NAFLD. In terms of mechanism, pachymaran may exert therapeutic effects through the *AMPK* pathway, resulting in changes in the expression of key proteins in the *AMPK* pathway. These insights may pave the way for novel therapeutic strategies in managing NAFLD.
